# Impact of body mass index on dietary habits and health behaviors in breast cancer patients

**DOI:** 10.3389/fonc.2026.1845987

**Published:** 2026-06-02

**Authors:** Agnieszka Bilska, Monika Radzimirska-Graczyk, Natalia Popierz-Rydlewska, Ewa Śliwicka, Marta Liszka, Piotr Nowaczyk, Wojciech Siejak, Anna Gogojewicz

**Affiliations:** 1 Department of Food and Nutrition, Poznan University of Physical Education, Poznań, Poland; 2 Department of Biochemistry, Poznan University of Physical Education, Poznań, Poland; 3 Department of Oncological Physiotherapy, Greater Poland Cancer Center, Poznań, Poland; 4 Breast Surgical Oncology Department, Greater Poland Cancer Center, Poznań, Poland

**Keywords:** body composition, body mass index, breast cancer, diet quality index, nutritional behaviors

## Abstract

**Introduction:**

Breast cancer incidence and mortality are projected to rise significantly by 2050, underscoring the need for integrated treatment strategies that address modifiable lifestyle factors. The aim of this study was to assess the impact of BMI on the dietary habits and health behaviors of women diagnosed with breast cancer.

**Methods:**

The study included 108 women (aged 31–65 years; mean 54.0 ± 9.15 years) recruited consecutively at the Breast Surgical Oncology Department of the Greater Poland Cancer Center, Poznań, Poland (from April to October 2023). Participants were divided into two groups based on BMI: Group 1 (within the normal range) and Group 2 (above the normal range). Dietary habits were assessed using the validated KomPAN questionnaire; health behaviors were assessed using the Health Behavior Inventory (HBI) by Juczyński.

**Results:**

Women with above-normal BMI presented a less favorable metabolic and behavioral profile, characterized by higher body fat percentage, greater visceral fat, shorter sleep duration, higher prevalence of tobacco use, lower leisure-time physical activity, and less regular meal patterns. Diet quality was higher in Group 1, with significantly greater consumption of vegetables, fruits, and fermented dairy products. Nutritional knowledge was insufficient in both groups, despite most women rating their own knowledge as adequate.

**Discussion:**

These findings underscore the need for targeted nutritional and behavioral interventions, encompassing dietary education, physical activity promotion, and sleep hygiene, integrated into standard oncological care, particularly for women with excess body weight. In line with recommendations from the American Society of Clinical Oncology (ASCO) and the European Society for Medical Oncology (ESMO), such interventions may reduce the risk of recurrence, improve prognosis, and enhance quality of life.

## 1 Introduction

Breast cancer is a major global public health challenge and underscores the need for integrated treatment strategies that incorporate multiple levels of clinical intervention alongside evidence−based preventive measures. According to a recent global analysis of trends across 185 countries, an estimated 2.3 million women were diagnosed with breast cancer in 2022, with 670,000 deaths recorded. If current rates continue, annual incidence is projected to reach 3.2 million cases and mortality 1.1 million deaths by 2050 (1, 2). These projections underscore the urgent need for integrated treatment and prevention strategies that address modifiable determinants of disease risk and outcomes. The determinants of breast cancer risk have been extensively investigated, with robust evidence demonstrating the significant influence of age, oral contraceptive use, menopausal status, genetic predisposition, and modifiable lifestyle factors, in particular body mass index (BMI), dietary habits, and physical activity, on disease development (3). Data from the World Health Organization (WHO) further highlight that the predominant modifiable risk factors currently include tobacco use, elevated body mass index (BMI), excessive alcohol consumption, insufficient intake of fruits and vegetables, and inadequate levels of physical activity (4).

Elevated BMI is a well-established modifiable risk factor for breast cancer, particularly in postmenopausal women, in whom adipose tissue becomes the primary site of estrogen synthesis. Excess adiposity promotes chronic low-grade inflammation and metabolic disturbances, including hyperinsulinemia, elevated IGF-1, and increased free estradiol bioavailability, that are recognized drivers of carcinogenesis and disease progression (3, 5). Importantly, the impact of BMI extends beyond diagnosis: women with excess body weight at the time of breast cancer diagnosis face a significantly higher risk of recurrence, poorer treatment response, and reduced overall survival compared with women of normal weight (5, 6).

Despite this, the relationship between BMI and specific dietary habits and health behaviors in women already diagnosed with breast cancer remains incompletely characterized. Evidence suggests that a substantial proportion of breast cancer patients do not adhere to recommended dietary guidelines and remain physically inactive after diagnosis — patterns that may be more pronounced in women with excess body weight (7–10).

Diets characterized by high intake of saturated fats, simple carbohydrates, processed meats, and sweetened beverages, along with low intake of fiber, fruit, and vegetables, contribute to chronic inflammation and metabolic disturbance, conditions that are particularly detrimental in women with elevated BMI (11–15). Conversely, improving dietary quality, reducing weight, and increasing physical activity are central components of both ASCO (16) and ESMO (17) recommendations to improve prognosis and quality of life after a breast cancer diagnosis.

Emerging evidence also points to the role of sleep disturbances and sleep duration as potential contributors to breast cancer risk (18, 19). Additionally, chronic psychological stress and lifestyle patterns shaped by prolonged emotional strain are increasingly recognized as relevant factors in both preventive strategies and clinical management (20). Despite growing evidence on the prognostic role of BMI in breast cancer, little is known about how BMI status differentiates dietary habits, nutritional knowledge, and health behaviors in women already diagnosed with the disease (21). Socioeconomic factors such as education and financial status are known to modulate these behaviors (22–24) and were examined as descriptive contextual variables. Addressing this gap is essential for designing targeted nutritional interventions within oncological care. Therefore, the aim of this study was to assess the impact of BMI on the dietary and health behaviors of women diagnosed with breast cancer.

## 2 Material and methods

### 2.1 Participants and study design

**Figure 1:**
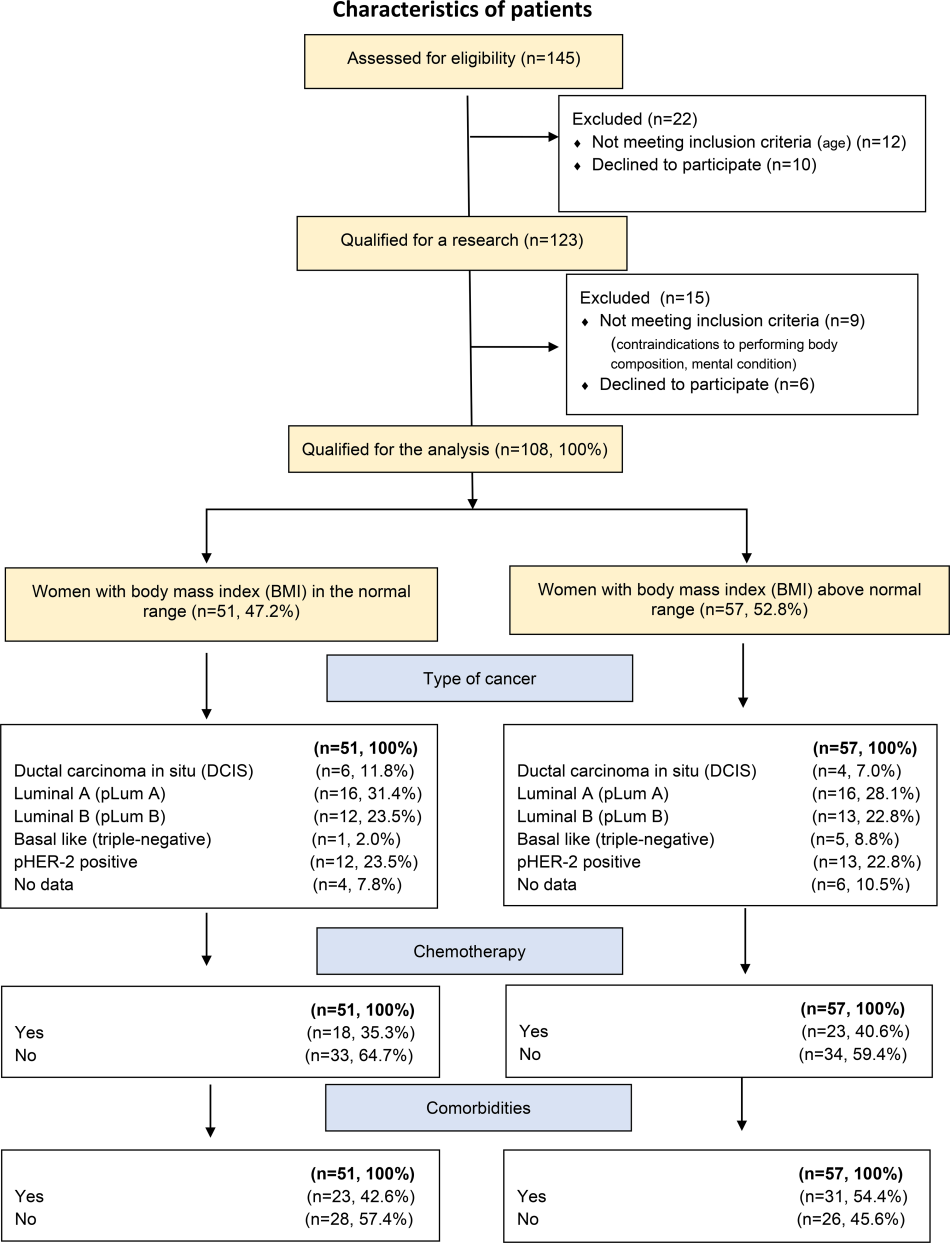
The exact characteristics of the patients.

The research was conducted in the Breast Surgical Oncology Department of the Greater Poland Cancer Center in Poznań, Poland. Consecutive sampling was used: all women admitted to the department between April and October 2023 who met the eligibility criteria, had not received formal nutritional education prior to the study, and provided written informed consent were invited to participate. The study included 108 women aged 31 to 65 years (mean age 54.0 ± 9.15 years) diagnosed with breast cancer. Participants were asked to report their dietary and lifestyle habits as they were prior to their cancer diagnosis. For comparative analysis, respondents were divided into two groups based on their body mass index (BMI): Group 1 — women with BMI within the normal range, and Group 2 — women with BMI above the normal range. The flow diagram of the study selection process is presented in **Figure 1**.

### 2.2 Inclusion and exclusion criteria

The study involved hospitalized patients diagnosed with breast cancer. Only women between 18 and 65 years of age who had not received formal nutritional education prior to participation were invited to the study. Exclusion criteria concerned the participants’ mental or physical condition, as well as the inability to perform body composition analysis due to the presence of metal implants in the body. Women over 65 years of age were also excluded from the study.

### 2.3 Nutritional status

#### 2.3.1 Anthropometric measurements

All anthropometric measurements were conducted in the morning, in a fasting state, by the same specialist (certified nutritionist). Body mass and height were measured using a certified digital medical grade scale and a mechanical measuring rod (WPT 60/150.O, Radwag, Radom, Poland), with an accuracy of 0.1 kg for weight and 0.5 cm for height, respectively. Body composition was assessed using the bioimpedance method, using the TANITA BC-420 analyzer (Tokyo, Japan) with GMON Professional software version 3.4.2 (Medizin and Service GmbH, Chemnitz, Germany). Measurements were conducted strictly in accordance with the recommended conditions (25), as previously described (26). Body mass index (BMI) was calculated as body mass (kg) divided by height squared (m²), according to the standard WHO formula. Participants were classified as having a normal BMI (18.5–24.9 kg/m²) or an above-normal BMI (≥25.0 kg/m²).

#### 2.3.2 Instruments

Two research tools, Juczyński’s Health Behavior Inventory (HBI) and the KomPAN questionnaire, were used to collect data on health behaviors.

Health behaviors were assessed using the Health Behavior Inventory (HBI) by Juczyński (27). The HBI questionnaire contains 24 statements describing health-related behaviors, divided into 4 categories: proper nutritional habits, preventive behaviors, health practices, and positive mental attitude. The statements were rated using a five-point response scale: almost never, rarely, occasionally, often, almost always, with points assigned from 1 to 5. The points obtained were summarized. The overall index of the intensity of health behaviors measured by the HBI ranges from 24 to 120 points. The higher the score, the greater the intensity of declared health behaviors. The questionnaire is intended for use with adult populations. The obtained number of points was converted into a sten scale. Low scores range from 24-77, average scores range from 78-91, and high scores range from 92-120. The reliability of the HBI is Cronbach’s α = 0.85, and its four subscales range from 0.60 to 0.65.

Dietary views and habits were studied using a validated Dietary Habits and Beliefs Questionnaire for people aged 16 to 65 years (KomPAN), developed by the Behavioral Nutrition Team of the Committee for Human Nutrition Science of the Polish Academy of Sciences. The self-administered version of the questionnaire (SA-Q) was used in the study. It consists of four parts grouped thematically with questions (28): A. Dietary habits B. Frequency of food consumption C. Views on food and nutrition D. Lifestyle and personal data. The study used a complete set of questions from section B to comprehensively characterize the frequency of food consumption by respondents. Based on 24 items, two dietary indices were created: the Prohealthy Diet Index (pHDI-10) (including 10 food products: questions 23, 25, 31–33, 37, 38, 40, 42, and 43) with a range of 0–20 points, and the Unhealthy Diet Index (nHDI) (14 products: questions 22, 24, 26–29, 34–36, 44, 46, 51–52 and 54) with a range of 0–28 points. The sum of consumption frequencies was used to classify each respondent into one of two groups of intensity of dietary traits, expressed in frequency per day: low (pHDI: 0.00–6.66; nHID: 0.00–9.33), moderate (pHID: 6.67–13.33; nHID: 9.34–18.66) and high (pHID: 13.34–20.00; nHID: 18.67–28.00). Additionally, the frequency of consumption of other product groups was examined (9 products: 30, 39, 41, 45, 47–50 and 53). For the diet index, the following intensity ranges of dietary traits expressed in points were adopted: low 0-33, moderate 34-66, and high 67-100. Food frequency consumption was evaluated in six categories (from ‘never’ (1) to ‘a few times a day’ (6)), assessing the habitual consumption of 24 food items. For each food item, the frequency consumption categories were converted to values reflecting daily frequency consumption (range: 0 to 2 times a day).

Part C of the questionnaire was used to assess nutritional knowledge. Part C consists of 25 statements related to food and nutrition (statements 55 to 79). Respondents can choose one of three answers: “True,” “False,” or “Hard to say.” In this study, the second proposed method of recoding responses was applied. Each statement was assigned 1 point for a correct answer (“True” or “False”) and 0 points for an incorrect answer or “Hard to say,” after which the points were summed. This approach evaluates and interprets only the correctly provided responses. Its advantage lies in its strong ability to differentiate respondents according to their level of nutrition knowledge. Based on the total score, respondents’ knowledge levels were classified as follows: insufficient (0–8 points), sufficient (9–16 points), and good (17–25 points).

### 2.4 Statistical analysis

All analyses were conducted using the Statistica 13.3 software package (TIBCO Software Inc., Palo Alto, CA, USA). The characteristics of the study participants were described using counts and percentages for qualitative variables. Differences between groups according to body mass index (BMI) were assessed using Pearson’s Chi-square test and the Maximum Likelihood Chi-square test, depending on sample size and expected frequencies. Results for anthropometric variables and HBI were presented as means with standard deviations, as well as medians and quartiles, stratified by groups. Comparisons between two BMI-based groups were performed using Student’s t-test or the Mann–Whitney U test, depending on the normality of distributions (evaluated with the Shapiro–Wilk test).

Food group consumption frequencies (KomPAN, part B) were expressed as daily intake frequencies by groups, and differences between BMI groups were analyzed using the Mann–Whitney U test. Dietary index results were reported as means with standard deviations, as well as medians and quartiles, stratified by groups. Comparisons between BMI groups were again performed using the Mann–Whitney U test due to the absence of normal distributions (verified with the Shapiro–Wilk test).

## 3 Results

**Table 1** presents the results of anthropometric and body composition measurements of women according to the body mass index. In the group of women with a BMI above the normal range, a markedly higher proportion of body fat was observed (mean 38.8% vs. 27.5%), accompanied by an elevated Visceral Fat Rating (mean 9.7 vs. 5.1). The proportion of fat−free mass in this group was more than 10% lower, amounting to 61.2% compared with 72.5% in Group 1. Total body water content was higher among women with a normal BMI (mean 50.2% vs. 43.1%).

**Table 1:** Selected anthropometric data of the study patients.

Variables	Group 1 (n=51)		Group 2* (n=57)		P-value
	Mean ± SD	Me (Q1-Q3)	Mean ± SD	Me (Q1-Q3)	
Age [years]	51.7 ± 8.90	53.3 (46.0-59.0)	56.8 ± 8.74	60.0 (51.0-65.0)	0.2531
BH [cm]	165.1 ± 6.13	164.0 (160.0-168.0)	164.8 ± 5.98	164.0 (160.0-170.0)	0.9287
BM [kg]	60.7 ± 5.85	60.0 (56.9-64.4)	80.9 ± 12.58	78.6 (72.6-87.1)	0.4290
BMI [kg/m^2^]	22.3 ± 1.96	22.5 (20.8-24.0)	29.9 ± 4.35	28.2 (26.7-31.8)	0.3564
FM [%]	27.5 ± 5.69	27.9 (24.0-31.6)	38.8 ± 5.22	37.8 (35.5-42.1)	0.3127
VFR [level]	5.1 ± 1.88	5.0 (4.0-7.0)	9.7 ± 2.84	9.0 (8.0-11.0)	0.8258
FFM[%]	72.5 ± 5.69	72.1 (68.4-76.0)	61.2 ± 5.21	62.2 (57.9-64.5)	0.3113
TBW [%]	50.2 ± 3.85	49.8 (47.6-51.9)	43.1 ± 2.81	43.5 (40.9-44.6)	0.6959

Data are presented as mean ± SD and interquartile ranges (Me (Q1-Q3)). BH, body height; BM, body mass; BMI, body mass index; FM, fat mass; VFR, visceral fat ranges; FFM fat-free mass; TBW, total body water;

* group 1 – women with BMI within the norm and group 2 – women with BMI above the norm.

**Table 2** presents the socio-demographic characteristics of the study group depending on the body mass index (BMI). In both study groups, the majority of women resided in rural areas, accounting for just under 42% among women with a BMI above the normal range and slightly more than 38% among women with a normal BMI. Respondents most commonly reported that their households consisted of two or three members and described their financial situation as average. A financial situation “above average” was indicated by 25.5% of women with a normal BMI and 17.8% of women in the second group. More than half of the women with a normal BMI (56.9%) assessed their household situation as “We live well — we can afford many things without special budgeting.” In contrast, 56.2% of respondents in the second group stated, “We live moderately — we manage day to day, but must save for major purchases.” Statistically significant differences at the level of p < 0.05 were observed in responses concerning employment status and educational attainment. As many as 80.4% of women with a normal BMI reported having stable employment. In the second group, this proportion was 47.4%, while an additional 43.8% were retired or receiving disability benefits. Nearly three times as many women in the first group had higher education (56.9% vs. 19.3%). Among women in the second group, secondary education was predominant (61.4%).

**Table 2:** Socio-economic characteristics [%].

Variables		Group 1 (n=51)	Group 2** (n=57)	P-value*
Q103. Place of residence	(1) : Village	38.30 (n=18)	41.82 (n=23)	0.7935
	(2) : Town under 20,000 inhabitants	12.77 (n=6)	18.18 (n=10)	
	(3) : Town from 20,000 to 100,000 inhabitants	25.53 (n=12)	20.00 (n=11)	
	(4) : City over 100,000 inhabitants	23.40 (n=11)	20.00 (n=11)	
	Missing data:	(n=4)	(n=2)	
Q104. Household size	1 2 3 4 5 6 7	9.80 (n=5) 39.22 (n=20) 31.37 (n=16) 15.69 (n=8) 1.96 (n=1) 0.00 (n=0) 1.96 (n=1)	15.79 (n=9) 36.84 (n=21) 22.81 (n=13) 10.53 (n=6) 8.77 (n=5) 1.75 (n=1) 3.51 (n=2)	0.4138
Q106. Financial situation	(1) : Below average	1.96 (n=1)	8.77 (n=5)	0.1526
	(2) : Average	72.55 (n=37)	75.44 (n=43)	
	(3) : Above average	25.49 (n=13)	15.79 (n=9)	
Q107. Household assessment	(1) : We live very poorly – we don’t have enough for even basic needs.	0.00 (n=0)	0.00 (n=0)	0.0838
	(2) : We live modestly – we have to be very frugal on a daily basis.	1.96 (n=1)	5.26 (n=3)	
	(3) : We live averagely – we have enough for everyday needs, but we have to save for major purchases.	39.22 (n=20)	56.15 (n=32)	
	(4) : We live well – we have enough for a lot without saving much.	56.86 (n=29)	33.33 (n=19)	
	(5) : We live very well – we can afford some luxury.	1.96 (n=1)	5.26 (n=3)	
Q108. Employment status	(1) : No, I’m retired or receiving a disability pension.	17.65 (n=9)	43.86 (n=25)	0.0019
	(2) : No, I’m on parental leave, unemployed, and I run a household.	1.96 (n=1)	3.51 (n=3)	
	(3) : Yes, but I work part-time.	0.00 (n=0)	5.26 (n=3)	
	(4) : Yes, I have permanent employment.	80.39 (n=41)	47.37 (n=27)	
	(5) : No, I’m studying.	0.00 (n=0)	0.00 (n=0)	
Q109. Education level	(1) : Primary education.	1.96 (n=1)	0.00 (n=0)	0.0001
	(2) : Vocational education.	3.92 (n=2)	19.30 (n=11)	
	(3) : Secondary education. or technical)	37.25 (n=19)	61.40 (n=35)	
	(4) : Higher (Bachelor’s, Engineering, Master’s)	56.86 (n=29)	19.30 (n=11)	

* red font indicates statistically significant differences between groups at the p <0.05 level, green indicates trend towards significance in the range 0.05<p<0.10; ** group 1 – women with BMI within the norm and group 2 – women with BMI above the norm; Q - question.

**Table 3** presents the results concerning women’s health behaviors and lifestyle depending on their body mass index. Approximately half of the women in both groups reported alcohol consumption, most commonly at a frequency of one to three times per month. When comparing the two groups, alcohol consumption was slightly more frequent among women with a normal BMI. A weekly drinking frequency was reported by 10% of women in Group 1 and 7% in Group 2. Drinking “several times per week” was declared exclusively by women with a normal BMI, representing 2% of this group. Among women who consumed alcohol, wine was the most frequently chosen beverage, reported by 73.9% of women in Group 1 and 58.6% in Group 2. Women with a BMI above the normal range more often selected beer (27.59% vs. 17.39%) and mixed drinks (13.79% vs. 8.7%) compared with women with a normal BMI.

**Table 3:** Selected health behaviors and lifestyle [%].

Variables		Group 1 (n=51)	Group 2** (n=57)	P-value*
Q54. Alcohol consumption frequency	(1) : Never	49.00 (n=25)	53.00 (n=30)	0.6087
	(2) : 1–3 times a month	39.00 (n=20)	40.00 (n=23)	
	(3) : Once a week	10.00 (n=5)	7.00 (n=4)	
	(4) : Several times a week	2.00 (n=1)	0.00 (n=0)	
	(5) : Once a day	0.00 (n=0)	0.00 (n=0)	
	(6) : Several times a day	0.00 (n=0)	0.00 (n=0)	
Q84. Preferred alcoholic beverage	(1) : Beer	17.39 (n=4)	27.59 (n=8)	0.5105
	(2) : Wine	73.91 (n=17)	58.62 (n=17)	
	(3) : Drinks	8.70 (n=2)	13.79 (n=4)	
	(4) : Hard liquor	0.00 (n=0)	0.00 (n=0)	
Q85. Current smoking	(1) : No	98.04 (n=50)	87.72 (n=50)	0.0937
	(2) : Yes	1.96 (n=1)	12.28 (n=7)	
Q86. Past smoking	(1) : No	68.63 (n=35)	43.86 (n=25)	0.0097
	(2) : Yes	31.37 (n=16)	56.14 (n=32)	
Q87. Weekday sleep duration	(1) : 6 or less hours/day	19.61 (n=10)	31.58 (n=18)	0.0365
	(2) : 7 or 8 hours/day	78.43 (n=40)	57.89 (n=33)	
	(3) : 9 or more hours/day	1.96 (n=1)	10.53 (n=6)	
Q88. Weekend sleep duration	(1) : 6 or less hours/day	13.73 (n=7)	19.30 (n=11)	0.1640
	(2) : 7 or 8 hours/day	82.35 (n=42)	68.42 (n=39)	
	(3) : 9 or more hours/day	3.92 (n=2)	12.28 (n=7)	
Q90. Physical activity at work	(1) : Low: more than 70% of time sitting	50.98 (n=26)	42.11 (n=24)	0.5532
	(2) : Moderate: about 50% of time sitting and about 50% of time moving	33.33 (n=17)	35.09 (n=20)	
	(3) : High: about 70% of time moving or physical work involving high exertion	15.69 (n=8)	22.81 (n=13)	
Q91. Leisure-time physical activity	(1) : Low: mostly sitting, watching TV, reading newspapers or books, light housework, walking 1–2 hours a week	17.65 (n=9)	35.09 (n=20)	0.0872
	(2) : Moderate: walking, cycling, gymnastics, gardening, or other light physical activity performed 2–3 hours a week	66.67 (n=34)	47.37 (n=27)	
	(3) : High: cycling, running, gardening, and other recreational sports requiring physical exertion performed more than 3 hours a week	15.69 (n=8)	17.54 (n=10)	

* red font indicates statistically significant differences between groups at the p <0.05 level, green indicates trend towards significance in the range 0.05<p<0.10; ** group 1 – women with BMI within the norm and group 2 – women with BMI above the norm; Q - question.

Statistically significant differences at the level of p < 0.05 were observed in responses regarding past tobacco use. As many as 56.14% of women with a BMI above the normal range reported having smoked in the past, compared with 31.37% in Group 1. The proportion of current smokers was six times higher in Group 2 (12.28% vs. 1.96%). Most women in both groups reported sleeping 7–8 hours per night on weekdays; however, this proportion was higher among women with a normal BMI (78.43% vs. 57.89%). Nearly one-third of women with a BMI above the normal range reported sleeping no more than 6 hours per night, and slightly more than 10% reported sleeping more than 9 hours. A similar pattern was observed for weekend sleep duration. Self-reported physical activity levels differed between work and leisure time. Moderate physical activity at work was declared by 33.33% of women in Group 1 and 35.09% in Group 2, whereas during leisure time, moderate activity was reported by 66.67% of women with a normal BMI and 47.37% of women with a BMI above the normal range. Notably, nearly 16% of women with a normal BMI reported high physical activity both at work and during leisure time. In comparison, 22.81% of women in the second group reported high physical activity at work, and 17.54% reported high activity during leisure time.

**Table 4** presents the results concerning women’s eating habits depending on their body mass index. The largest proportion of women in both groups reported consuming four meals per day. Statistically significant differences were observed in responses regarding meal regularity and snack consumption. Regular consumption of at least some meals was declared by 67% of women in Group 1 and 39% in Group 2. As many as 32% of women with a BMI above the normal range stated that they did not eat meals at fixed times. Women in both groups reported consuming snacks once or several times per day, most commonly fruit. This response was given by 86% of women in Group 1 and 70% in Group 2. Sweet snacks such as candies and chocolate bars were consumed by 42% of respondents in Group 1 and 33% in Group 2. Consumption of nuts, seeds, and kernels was declared by 47% of women with a normal BMI and 28% of women with a BMI above the normal range. Additionally, 5% of respondents in Group 2 reported consuming “other products,” most frequently ice cream, as snacks. Still water was the beverage most commonly consumed by respondents, reported by 90% of women in Group 1 and 88% in Group 2. Among women with a BMI above the normal range, carbonated water and flavored water were consumed by 28% and 11%, respectively, compared with 16% and 4% in Group 1.

**Table 4:** Selected eating habits.

Variables		Group 1 (n=51)	Group 2** (n=57)	P-value*
Q7. Number of meals during the day	(1): 1 meal	0.00 (n=0)	0.00 (n=0)	0.5554
	(2): 2 meals	6.00 (n=3)	5.00 (n=3)	
	(3): 3 meals	27.00 (n=14)	39.00 (n=22)	
	(4): 4 meals	49.00 (n=25)	46.00 (n=24)	
	(5): 5 meals or more	18.00 (n=9)	11.00 (n=6)	
Q8. Meal regularity	(1): No	18.00 (n=9)	32.00 (n=18)	0.0142
	(2): Yes, but only some	67.00 (n=34)	39.00 (n=22)	
	(3): Yes, all	16.00 (n=8)	30.00 (n=17)	
Q9. Snacking frequency	(1): Never	6.00 (n=3)	5.00 (n=3)	0.3551
	(2): 1–3 times a month	12.00 (n=6)	11.00 (n=6)	
	(3): Once a week	2.00 (n=1)	11.00 (n=6)	
	(4): Several times a week	25.00 (n=13)	16.00 (n=9)	
	(5): Once a day	25.00 (n=13)	33.00 (n=19)	
	(6): Several times a day	29.00 (n=15)	25.00 (n=14)	
Q10. Snack type (weekdays)	(10/1) : Fruits	86.00 (n=44)	70.00 (n=40)	0.0445
	(10/2) : Vegetables	20.00 (n=10)	32.00 (n=18)	0.1564
	(10/3) : Unsweetened dairy drinks and desserts, e.g. yogurt, cottage cheese, milk	29.00 (n=15)	21.00 (n=12)	0.3166
	(10/4) : Sweetened dairy drinks and desserts, e.g. homogenized cheeses, sweetened milk drinks, flavored milk	8.00 (n=4)	14.00 (n=8)	0.3089
	(10/5) : Sweet snacks, e.g. candies, cookies, cakes, chocolate bars, muesli bars, waffles	43.00 (n=22)	33.00 (n=19)	0.2946
	(10/6) : Salty snacks, e.g. crackers, breadsticks, chips, French fries	14.00 (n=7)	19.00 (n=11)	0.4400
	(10/7) : Nuts, almonds, seeds, pits	47.00 (n=24)	28.00 (n=16)	0.0413
	(10/8) : Other products, please specify which ones?	0.00 (n=0)	5.00 (n=3) (ice cream)	0.2823
Q12. Meat preparation method	(12/1) : Boiled	43.00 (n=22)	53.00 (n=30)	0.3242
	(12/2) : Stewed	45.00 (n=23)	42.00 (n=24)	0.7541
	(12/3) : Grilled	14.00 (n=7)	16.00 (n=9)	0.7642
	(12/4) : Baked	45.00 (n=23)	42.00 (n=24)	0.7541
	(12/5) : Fried	41.00 (n=21)	40.00 (n=23)	0.9305
	(12/6) : I do not eat meat	8.00 (n=4)	5.00 (n=3)	0.8790
Q17. Water type	(17/1) : I do not drink water	0.00 (n=0)	0.00 (n=0)	1.0000
	(17/2) : Still water	90.00 (n=46)	88.00 (n=50)	0.6840
	(17/3) : Sparkling water	16.00 (n=8)	28.00 (n=16)	0.1222
	(17/4) : Flavored water	4.00 (n=2)	11.00 (n=6)	0.3470

* red font indicates statistically significant differences between groups at the p <0.05 level; ** group 1 – women with BMI within the norm and group 2 – women with BMI above the norm; Q - question.

**Table 5** presents the mean values of the dietary indices. The intensity of pro−health dietary characteristics (pHDI−10) was low in both groups; however, the mean score was slightly higher in Group 1 (26.4) compared with Group 2 (23.3). Regardless of BMI category, the participants’ diets were characterized by a low intensity of unhealthy dietary features (nHDI−14). Additionally, the frequency of consumption of the components included in the Pro−Health Diet Index (pHDI−10) and the Non−Healthy Diet Index (nHDI−14) was analyzed.

**Table 5:** Diet quality index (based on the pHDI−10 and nHDI−14 components).

Variables	Group 1 (n=51)		Group 2* (n=57)		P-value
	Mean ± SD	Me (Q1-Q3)	Mean ± SD	Me (Q1-Q3)	
pHDI-10 points	26.4 ± 10.43	24.8 (19.7-33.8)	23.3 ± 8.92	23.4 (16.6-28.1)	0.1011
nHDI-14 points	14.5 ± 6.65	14.1 (9.9-19.0)	14.1 ± 6.96	12.6 (8.8-17.8)	0.4734

pHDI-10, Pro-Healthy-Diet-Index-10 nHDI-14, Non-Healthy-Diet-Index-14; * group 1 – women with BMI within the norm and group 2 – women with BMI above the norm.

Based on the data presented in **Table 6** for the pHDI−10, women with a normal BMI more frequently selected foods with health−promoting properties than women in the second group. These included, among others, vegetables and fruits. The differences in consumption frequency of these products between the groups were statistically significant. Fruit consumption frequency was 1.36 vs. 1.10, and vegetable consumption frequency was 1.15 vs. 0.88 in Groups 1 and 2, respectively. Furthermore, women with a normal BMI more often consumed fermented milk beverages and fresh cheese curd products compared with women in the second group. Analysis of the consumption frequency of the components of the Non−Healthy Diet Index did not reveal statistically significant differences between the groups. However, women in Group 2 more frequently consumed cold meats, smoked sausages, hot dogs, red meat, and sweetened beverages. Additionally, only women in this group reported consuming energy drinks. When analyzing the consumption frequency of other product categories, a trend toward statistical significance (p < 0.10) was observed for canned vegetables and vegetable or vegetable−fruit juices. Both product groups were consumed more frequently by women in Group 1. The consumption frequencies were 0.26 vs. 0.18 and 0.32 vs. 0.23, respectively.

**Table 6:** Food frequency consumption [times/day] according to KomPAN part B.

No.	Questionnaire items	Group 1 (n=51)	Group 2** (n=57)	P-value*	Total
		Mean	Mean		Mean
*Pro−Healthy Diet Index* (pHDI−10) and its components in the KomPAN questionnaire					
23	Wholemeal bread	0.44	0.58	0.6581	0.51
25	Buckwheat, oats, wholegrain pasta, other coarse-groundgroats	0.26	0.24	0.3579	0.25
31	Milk (including flavored milk, cocoa, coffee with milk)	0.46	0.51	0.7346	0.49
32	Fermented milk beverages, e.g. yogurts, kefir (natural or flavored)	0.50	0.38	0.1120	0.44
33	Fresh cheese curd products (including homogenized cheese, cottage cheese desserts)	0.44	0.37	0.2741	0.40
37	White meat, e.g. chicken, turkey, rabbit	0.34	0.36	0.3593	0.35
38	Fish	0.15	0.14	0.7770	0.14
40	Pulse-based foods, e.g. beans, peas, soy, lentils	0.18	0.12	0.8389	0.15
42	Fruit	1.36	1.10	0.0472	1.22
43	Vegetables	1.15	0.88	0.0242	1.01
*Unhealthy Diet Index* (nHDI−14) and its components in the KomPAN questionnaire					
22	White bread, e.g. wheat, rye, mixed wheat and rye, toasted bread, rolls, croissants	1.00	0.87	0.6028	0.93
24	White rice, white pasta, fine-ground groats, e.g. semolina, couscous	0.30	0.24	0.6739	0.27
26	Fast foods, e.g. fries, hamburgers, pizza, hot dogs, casseroles	0.04	0.05	0.6142	0.05
27	Fried meat or flour foods	0.24	0.26	0.7682	0.25
28	Butter as an addition to bread or dishes, for frying, baking, etc.	0.98	0.95	0.828	0.70
29	Lard as an addition to bread or dishes, for frying, baking, etc.	0.06	0.06	0.7184	0.06
34	Cheese (including processed cheese, blue cheese)	0.40	0.36	0.6082	0.38
35	Cold meats, smoked sausages, hot-dogs	0.33	0.43	0.2303	0.38
36	Red meat, e.g. pork, beef, veal, mutton, lamb, game	0.16	0.21	0.2021	0.19
44	Sweets, e.g. candies, cookies, cakes, chocolate bars, muesli bars, other confectionery products	0.46	0.37	0.2926	0.41
46	Tinned meat	0.01	0.02	0.2926	0.01
51	Sweetened beverages carbonated or non-carbonated such as Coca Cola, Pepsi, Sprite, Fanta, orangeade, lemonade	0.04	0.06	0.8543	0.05
52	Energy drinks, e.g. 2 KC, Black Horse, Red Bull, Burn, Shot and other	0.00	0.02	0.1004	0.01
54	Alcoholic beverages	0.05	0.03	0.5690	0.04
*Frequency of consumption of other food groups*					
30	Oils, margarines, or butter–margarine blends	0.30	0.36	0.6097	0.33
39	Eggs	0.32	0.38	0.3748	0.35
41	Potatoes (excluding fries and chips)	0.39	0.42	0.4556	0.40
45	Instant soups or ready−made canned soups	0.01	0.02	0.3337	0.01
47	Canned vegetables	0.26	0.18	0.0510	0.25
48	Fruit juices	0.39	0.36	0.9242	0.37
49	Vegetable or vegetable−fruit juices	0.32	0.23	0.0643	0.27
50	Sweetened hot beverages	0.92	0.70	0.3362	0.80
53	Mineral or table water	1.61	1.61	0.9640	1.61

* red font indicates statistically significant differences between groups at the p <0.05 level, green indicates trend towards significance in the range 0.05<p<0.10; ** group 1 – women with BMI within the norm and group 2 – women with BMI above the norm.

**Figure 2:**
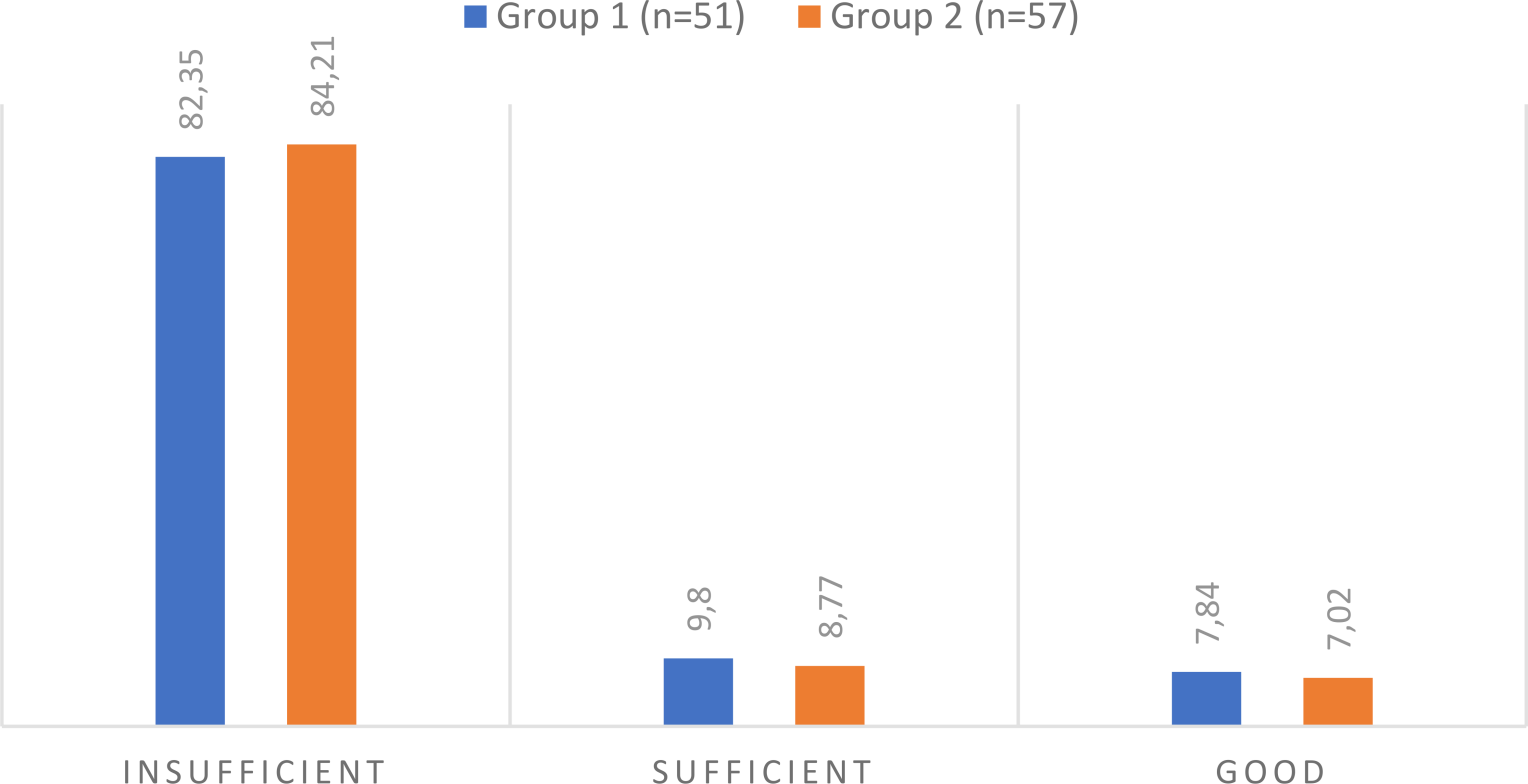
Assessment of nutrition knowledge among patients using KomPAN questionnaire part C [%]. Level of nutritional knowledge: insufficient (0–8 points), sufficient (9–16 points), good (17–25 points) (p-value 0.9672).

**Figure 3:**
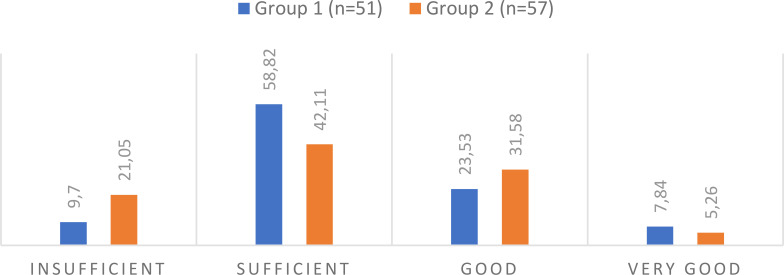
Subjective assessment of the level of nutritional knowledge using KomPAN questionnaire [%], question 93 (p-value 0.1988).

Analysis of the KomPAN questionnaire results (Part C) showed that both groups of women demonstrated an insufficient level of nutrition knowledge (**Figure 2**). The proportion of women with insufficient knowledge was slightly higher in Group 2 than in Group 1 (84.21% vs. 82.35%). Notably, the participants’ subjective assessment of their nutrition knowledge was considerably higher (**Figure 3**). Responses to Question 93 of the KomPAN questionnaire indicated that 42.11% of women in Group 2 and 58.82% in Group 1 believed they possessed sufficient nutrition knowledge, while an additional 31.58% and 23.53%, respectively, rated their knowledge as good. Fewer than 10% of women with a normal BMI and 21% of those with a BMI above the normal range assessed their knowledge as insufficient.

**Figure 4:**
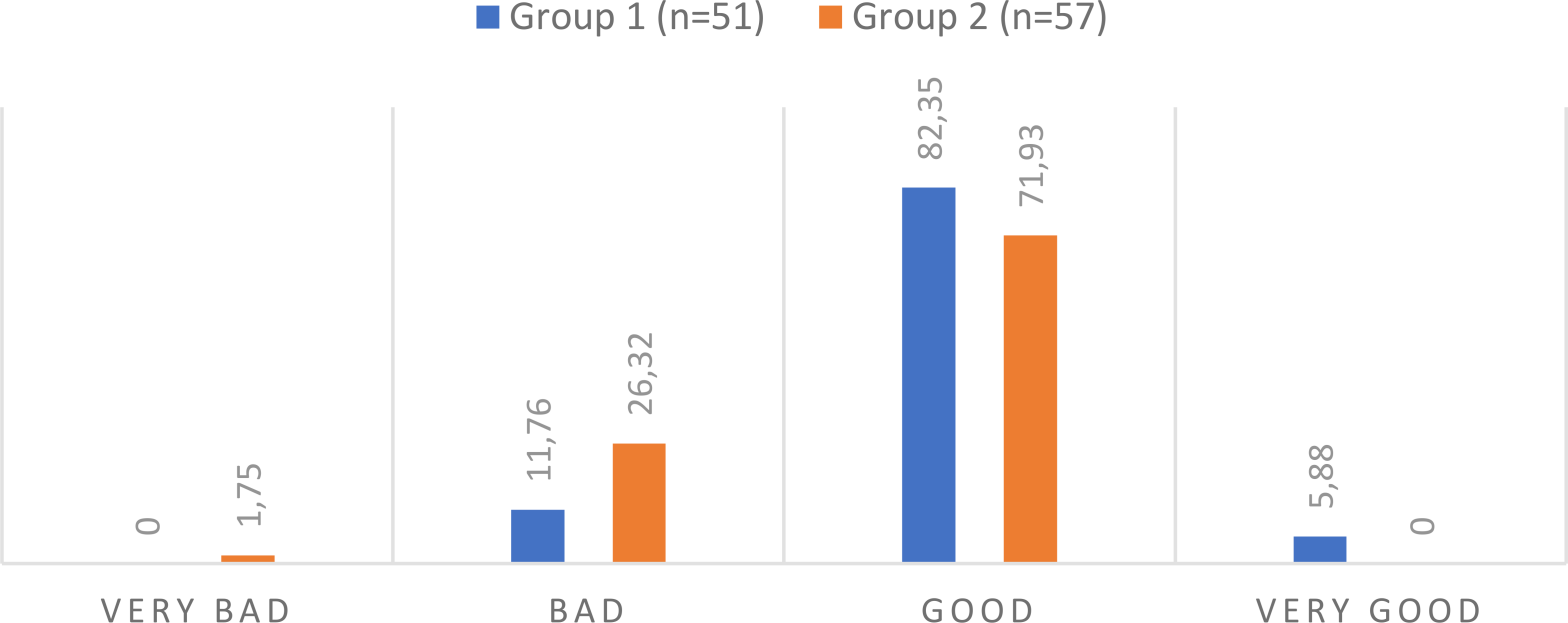
Self-assessment of nutrition using KomPAN questionnaire [%], question 94 (p-value* 0.0266). * red font indicates statistically significant differences between group at the p <0.05 level.

Subjective evaluation of dietary habits was also examined based on responses to Question 94 of the KomPAN questionnaire (**Figure 4**). Statistically significant differences were observed at the level of p < 0.05. Most respondents, regardless of group, declared that their dietary habits were good; however, this response was more frequent among women with a normal BMI (82.35% vs. 71.93%). Only women in Group 1 described their dietary habits as “very good,” accounting for 5.88% of respondents. In contrast, 1.75% of women in Group 2 reported that their dietary habits were “very poor. “**Table 7** presents the results of 108 women according to the body mass index. Preventive behavior (p=0.0840) and positive mental attitude (p=0.0536) were higher in women with BMI above normal range. However, proper eating habits and health practices were lower in this group. These differences resulted in a higher Health Behavior Index observed in women with normal range BMI.

**Table 7:** Health behavior inventory (HBI) by Juczyński.

Variables	Group 1 (n=51)		Group 2** (n=57)		P-value*
	Mean ± SD	Me (Q1-Q3)	Mean ± SD	Me (Q1-Q3)	
PEH	23.6 ± 3.70	24.0 (15.0-30.0)	22.5 ± 3.98	22.0 (13.0-30.0)	0.1556
PB	22.7 ± 4.00	22.0 (15.0-30.0)	23.9 ± 4.64	25.0 (14.0-30.0)	0.0840
PMA	22.5 ± 3.92	22.0 (14.0-30.0)	23.9 ± 3.36	24.0 (17.0-30.0)	0.0536
HP	21.4 ± 3.57	22.0 (15.0-30.0)	21.3 ± 3.76	21.0 (12.0-28.0)	0.9951
HBI	90.3 ± 10.51	88.0 (62.0-117.0)	88.4 ± 10.25	93.0 (70.0-109.0)	0.5038

PEH, Proper eating habits; PB, Preventive behavior; PMA, Positive mental attitude; HP, Health practices; HBI, Health behavior index.;* green font indicates trend towards significance in the range 0.05<p<0.10; ** group 1 – women with BMI within the norm and group 2 – women with BMI above the norm.

## 4 Discussion

The findings of the present study confirm that lifestyle and dietary behaviors among women diagnosed with breast cancer differ significantly depending on their nutritional status, expressed by BMI. Consistent with current epidemiological evidence, excess body weight is one of the most important modifiable risk factors for breast cancer, particularly in the postmenopausal period (3, 5, 16, 29). Women with a BMI above the normal range exhibited a significantly higher proportion of body fat (38.8% vs. 27.5%) and a higher visceral fat rating (9.7 vs. 5.1), which aligns with observations indicating that obesity promotes chronic inflammation and metabolic disturbances that may increase the risk of cancer development, including breast cancer (6, 7, 18, 30, 31). In postmenopausal women, who constituted a significant part of the study population aged 49–63 years, adipose tissue becomes the primary site for estrogen biosynthesis. This process is catalyzed by aromatase-mediated conversion of adrenal androgens, which is further enhanced by pro-inflammatory cytokines such as TNF-alpha and IL-6, typically upregulated in obese fat (32, 33). Furthermore, visceral fat significantly increases local and systemic levels of estrogens and insulin-like growth factors, which are known to promote cancer development and potentially hinder favorable clinical outcomes (6). Although this link is most pronounced after menopause, visceral obesity also contributes to a pro-oncogenic environment in premenopausal women by inducing hyperinsulinemia. This condition lowers sex hormone-binding globulin (SHBG) levels, thereby increasing the bioavailability of free estradiol and promoting mitogenic signaling via the insulin/IGF-1 pathway (34). Therefore, the increased prevalence of visceral adiposity observed in Group 2 may not only increase the risk of carcinogenesis but also worsen prognosis and raise the risk of disease recurrence.

### 4.1 Socio−demographic differences and BMI

The results indicate that women with a normal BMI more frequently had higher education and stable employment. This relationship is consistent with literature emphasizing that higher socioeconomic status is associated with better health awareness, healthier dietary choices, greater physical activity, and more frequent use of preventive healthcare (20, 22, 35, 36). Lower educational attainment and poorer financial status—more common among women with a BMI above the normal range—may contribute to less favorable dietary choices and irregular meal patterns. These factors may indirectly influence breast cancer risk and quality of life after diagnosis. Similar associations have been reported in population studies, where low socioeconomic status was linked to a higher risk of obesity and poorer diet quality (3, 23, 24, 37, 38).

The observed differences in dietary behaviors between BMI groups should be interpreted with caution, as the two groups also differed significantly in educational attainment and employment status — factors known to independently influence dietary choices and health behaviors (22–24). These socioeconomic variables may act as potential confounders, meaning that part of the observed variation in diet quality could reflect socioeconomic disadvantage rather than BMI status per se. Given the multidimensional, wide-ranging scope of the KomPAN questionnaire, which encompasses a large number of food items and behavioral variables, a single comprehensive multivariable analysis was not feasible. The findings should therefore be considered exploratory.

### 4.2 Sleep, stress, and lifestyle as risk factors

Women with a BMI above the normal range more frequently reported shorter sleep duration. Cohort studies indicate that short sleep (<6 hours) and circadian rhythm disturbances may increase breast cancer risk through dysregulation of the hypothalamus-pituitary-adrenal (HPA) axis, altered melatonin secretion, increased oxidative stress, and impaired glucose metabolim (19, 39–42). This group also showed a higher prevalence of current and past tobacco use. Tobacco smoking is a major modifiable risk factor for breast cancer (16) and its higher prevalence among women with excess body weight may further worsen their health profile. Leisure−time physical activity was markedly lower among women with a BMI above the normal range, consistent with evidence that individuals with excess weight are less likely to engage in regular physical activity. This is particularly important, as physical activity plays a key role in both prevention and improved prognosis among women with breast cancer (8, 43–45).

### 4.3 Dietary behaviors, diet quality, and nutrition knowledge

Analysis of the KomPAN results showed that women with a normal BMI more frequently consumed health−promoting foods such as vegetables, fruits, fermented milk beverages, and fresh cheese curd products. According to the literature, a diet rich in fiber, antioxidants, and phytochemicals may reduce breast cancer risk by lowering oxidative stress, modulating gut microbiota, and influencing estrogen metabolism (7, 11–15). In contrast, women with a BMI above the normal range more frequently consumed red meat, processed meats, sweetened beverages, and highly processed foods. These products are rich in saturated fats, salt, simple sugars, and pro−inflammatory compounds that may increase cancer risk through induction of chronic inflammation and metabolic disturbance (18, 46–49). It is noteworthy that the intensity of pro−health dietary characteristics (pHDI−10) was low in both groups, indicating a general need for nutrition education among women with breast cancer.

The KomPAN Part C results showed that most women demonstrated insufficient nutrition knowledge, with a slightly higher proportion in the group with a BMI above the normal range. At the same time, subjective assessments of nutrition knowledge were substantially overestimated relative to actual test results, suggesting overconfidence in perceived health competence. This discrepancy may reflect a range of factors, including limited health literacy, a tendency to overestimate one’s own competence when assessed in a clinical setting, and response bias inherent in self-report instruments. Overconfidence in one’s own nutritional knowledge, which may be explained by the cognitive pattern described in the literature as the Dunning–Kruger effect (50, 51), may hinder engagement with health education regardless of its underlying cause (52).

### 4.4 Health behaviors

The results of the Health Behavior Inventory (HBI) indicate that women with a normal BMI exhibited a higher overall level of health behaviors (HBI), particularly in the domains of proper eating habits (PEH) and health practices (HP). Interestingly, women with a BMI above the normal range scored higher in preventive behaviors (PB) and positive mental attitude (PMA). This may reflect a “health mobilization effect” following a cancer diagnosis, as individuals at higher metabolic risk may be more motivated to seek psychological support and adopt preventive strategies (53, 54). Comparable findings were reported in earlier research, where proper eating habits (PEH) and overall HBI scores were significantly higher among women whose physical activity levels met WHO recommendations (55).

Nutritional and behavioral interventions should constitute an integral component of care for women with breast cancer. According to ASCO and ESMO recommendations, lifestyle modification—including weight reduction, increased physical activity, improved diet quality, and better sleep hygiene—may improve prognosis, reduce recurrence risk, and enhance quality of life (8, 54, 56).

### 4.5 Limitations

Several limitations of this study should be considered. First, the sample size of 108 women from a single clinical center limits statistical power and the generalizability of the findings. Second, retrospective recall of pre-diagnosis lifestyle data may introduce recall bias. Third, the two BMI groups differed significantly in socioeconomic characteristics, particularly educational attainment (p < 0.001) and employment status (p = 0.002). These variables are known determinants of dietary behavior and may act as residual confounders; the observed differences in diet quality between groups may therefore partly reflect socioeconomic disparities rather than BMI status alone. No multivariable analysis was conducted — given the sample size, the exploratory nature of the study, and the wide, multidimensional scope of the KomPAN instrument, a single coherent multivariable model encompassing all nutritional variables was not appropriate. Fourth, the large number of statistical comparisons increases the risk of type I error; no correction for multiple testing was applied, and findings with p-values near the 0.05 threshold should be interpreted with appropriate caution. Fifth, self-reported data from the KomPAN and HBI questionnaires may be subject to social desirability bias, which may partly account for the gap between subjective self-assessments and objectively measured nutritional knowledge. Finally, the cross-sectional design precludes causal inference.

## 5 Conclusions

The findings of this study indicate that BMI status significantly differentiates lifestyle, dietary habits, and health behaviors among women with breast cancer. Women with BMI above the normal range presented a less favorable metabolic and behavioral profile, characterized by higher body fat percentage, greater visceral fat, shorter sleep duration, higher prevalence of tobacco use, lower leisure-time physical activity, and less regular meal patterns. Diet quality was higher among women with normal BMI, who more frequently consumed vegetables, fruits, and fermented dairy products, while women with above-normal BMI more often chose processed foods, red meat, and sweetened beverages.

Nutritional knowledge was insufficient in both groups, despite most women rating their own knowledge as sufficient or good. This discrepancy, together with the socioeconomic differences observed between groups, underscores the need for targeted nutritional and behavioral interventions integrated into standard oncological care — encompassing dietary education, physical activity promotion, and sleep hygiene — particularly for women with excess body weight. In line with ASCO and ESMO recommendations, such interventions may reduce the risk of recurrence, improve prognosis, and enhance quality of life.

## Data Availability

The raw data supporting the conclusions of this article will be made available by the authors, without undue reservation.
